# The potential for large-scale genome editing in finfish aquaculture via germline stem cell culture and transplantation

**DOI:** 10.3389/fgene.2026.1924271

**Published:** 2026-10-26

**Authors:** Mari Raudstein, Alex Kojo Datsomor, Robert Stewart, Mike McGrew, Clémence Fraslin, Diego Crespo, Lene Kleppe

**Affiliations:** 1 The Roslin Institute and Royal (Dick) School of Veterinary Studies, University of Edinburgh, Midlothian, United Kingdom; 2 Institute of Marine Research, Reproduction and Developmental Biology Research Group, Bergen, Norway; 3 Benchmark Genetics Norway AS, Bergen, Norway

**Keywords:** aquaculture, CRISPR/Cas, genome editing, germ cell transplantation, germline stem cells, surrogate broodstock technology, Atlantic salmon

## Abstract

Genome editing has emerged as a promising tool to complement selective breeding programmes and accelerate genetic gain in aquaculture. Although CRISPR-based genome editing has been successfully applied in several economically important fish species, its application at an industry-relevant scale remains challenging due to limitations of current delivery methods, which primarily rely on manual embryo microinjection. This approach is labour-intensive, technically demanding, and frequently results in high levels of mosaicism in founder individuals, necessitating additional generations of breeding to establish stable genotypes. In this mini review, we discuss the potential of combining germline stem cell (GSC) culture and transplantation approaches as an alternative platform for further improved genome editing in fish. By enabling genetic changes to be introduced, validated, and expanded *in vitro* prior to germline transmission, this approach has the potential to improve scalability, reduce mosaicism, and shorten breeding timelines. Although several technical challenges remain, continued progress in GSC culture and transplantation methodologies could facilitate the practical implementation of tailored genome editing for improved genetic gain, fish health, and welfare in aquaculture.

## 1 Introduction

Aquaculture plays an increasingly important role in supplying the global population with nutritious food. Although the sector has grown steadily over recent decades, several biological and technological challenges still limit its ability to meet future seafood demand in a sustainable and fish welfare-friendly way. Compared with terrestrial livestock farming, aquaculture is a relatively young industry with considerable potential for genetic improvement of production fish through selective breeding (Boudry et al., 2021; Houston et al., 2020). Nevertheless, challenges such as long generation intervals in certain economically important fish species, and the complexity of selectively targeting multiple traits with potential trade-offs might hamper genetic gains from traditional breeding programmes. More recently, genome editing has been proposed as a complementary approach that could accelerate the precise introduction of valuable traits related to reproduction and sterility, growth, disease resistance, and overall fish health and welfare (Gratacap et al., 2019; Yang et al., 2022).

The discovery of CRISPR/Cas-based genome editing has led to advances in functional genomics across a wide range of aquaculture fish species such as Atlantic salmon (Almeida et al., 2022; Andersson et al., 2024; Crespo et al., 2024; Datsomor et al., 2019; Skaftnesmo et al., 2021; Wargelius et al., 2016), rainbow trout (Fujihara et al., 2022), Nile tilapia (Jiang et al., 2017; Yang et al., 2022), common carp (Chen et al., 2019; Zhong et al., 2016), and channel catfish (Coogan et al., 2022). However, despite these successes, the implementation of genome editing at an industry-relevant scale remains challenging due to both technical limitations and the globally uncertain regulatory and commercial landscape. Currently, *in vivo* genome editing in fish is primarily achieved by manual microinjection into newly fertilised eggs (i.e., embryos), a labour-intensive process that limits scalability. In addition, mosaicism is highly prevalent in founder F0 fish, making it necessary to breed edited individuals to obtain fish lines carrying the intended genetic mutation. For species with long generation intervals this represents a significant bottleneck.

One potential way to facilitate more refined and scalable genome editing is via germline stem cell (GSC) *in vitro* culture followed by transplantation into sterile surrogate broodstock. In a cell culture system, genetic edits can be validated prior to introduction into sterile surrogates. This approach could bypass multigenerational breeding, allowing the direct production of gametes carrying the desired genetic modification within a single generation. With a particular focus on Atlantic salmon, this mini review evaluates the challenges of current genome editing approaches and discusses how robust GSC culture systems may serve as a foundation for industry-scale genome editing in finfish aquaculture.

## 2 Current challenges in fish genome editing

Despite major advances in genome editing over the past decade, the application of this technology in aquaculture species remains mostly limited to experimental and proof-of-concept studies. A few genome-edited fish species are available as commercial market products in Japan (Japan embraces CRISPR-edited fish, 2022); however, both technical and regulatory challenges hinder its wider implementation at an industry scale. While recognising the importance of regulatory challenges, this mini review focuses on the technical challenges only, as regulatory frameworks vary substantially between countries and continue to evolve rapidly.

Genome editing in fish is primarily performed by manual microinjection of CRISPR/Cas components into fertilised eggs at an early developmental stage, preferably the one-cell stage (Figure 1A). This delivery method is labour-intensive and requires high technical accuracy, which limits throughput and scalability. In addition, the procedure of microinjection can lead to mortality due to mechanical damage to the embryo/egg, but also due to potential toxicity of the reagents introduced. Furthermore, genome-edited F0 founder fish often exhibit a high degree of mosaicism. Mosaicism occurs when embryos are microinjected at multicellular stages and/or the editing is slow or incomplete, leading to individuals with a mixed population of edited and unedited (wild-type) cells (Mehravar et al., 2019). Incomplete genome editing may occur due to variation in the guide RNA (gRNA)’s ability to produce mutations in the genomic DNA (i.e., gRNA efficiency), as some genomic target sites are more amenable to editing than others. Although several computational tools exist to predict gRNA efficiency, their accuracy remains insufficient to prevent mosaic outcomes (Uribe-Salazar et al., 2022). In addition to the presence of unedited cells, edited cells within the same individual may carry different mutations at different frequencies. These may include frameshift mutations that disrupt gene function, as well as in-frame mutations that retain partial or full protein activity. Consequently, phenotypic outcomes in F0 founder fish are generally difficult to interpret. Due to these limitations, genome-edited F0 founder male fish (that often mature earlier than females) are typically crossed with wild-type females to generate defined heterozygous genotypes in F1 offspring (Figure 1B, upper panel). Further breeding to the F2 generation is required to obtain biallelic mutant fish among the offspring. For species with long generation intervals, such as Atlantic salmon (∼3–4 years), this significantly increases the time required to validate and implement genome-edited traits in a broodstock population. Alternatively, crossing F0 founders enables the production of biallelic genome-edited fish in the F1 generation, although certain genome-edited individuals may carry different mutation variants in the two alleles (Figure 1B, lower panel).

**FIGURE 1:**
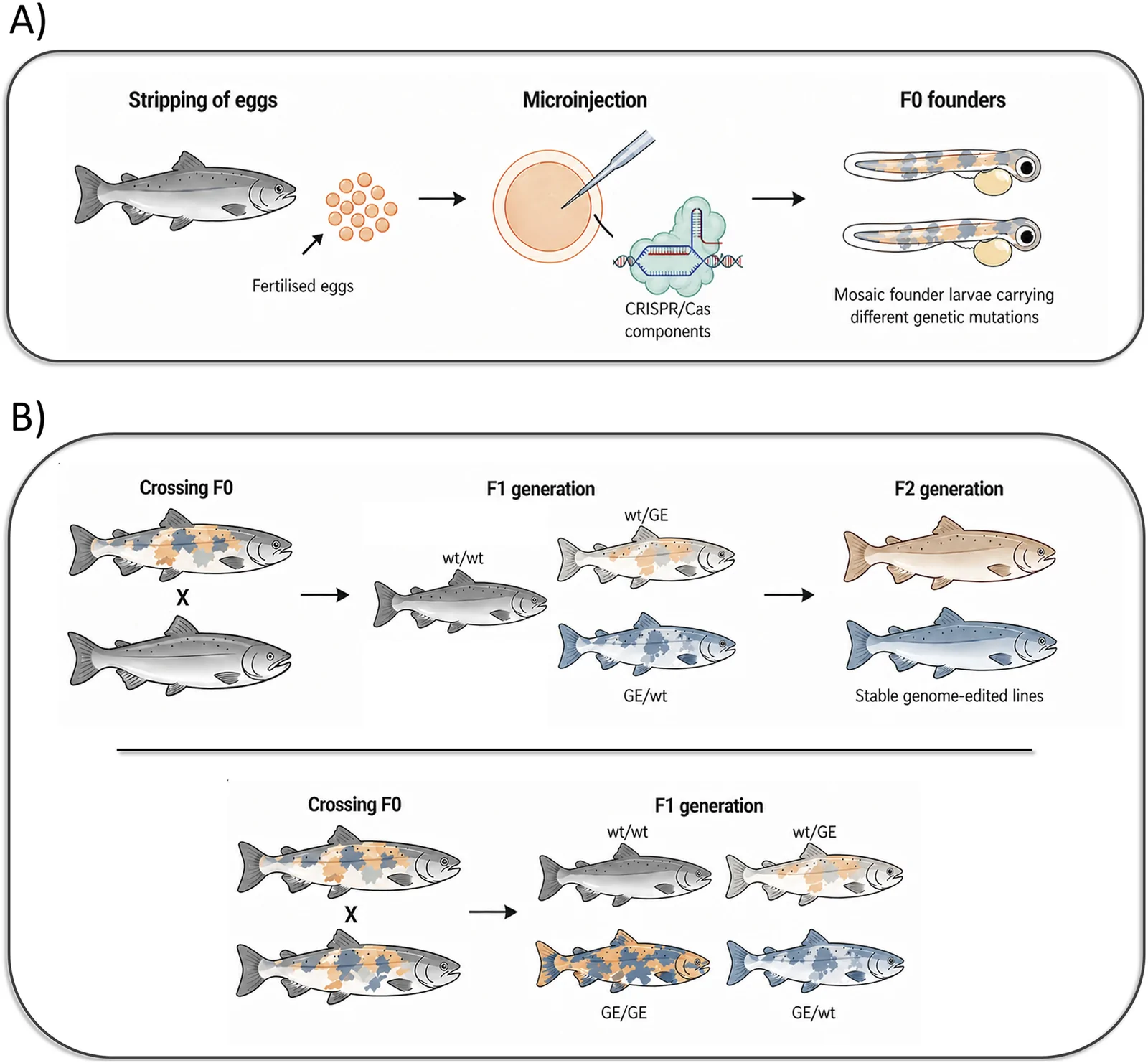
Current *in vivo* genome editing workflow in Atlantic salmon. **(A)** CRISPR/Cas components targeting the gene(s) of interest are microinjected into fertilised eggs, producing mosaic F0 founder fish carrying different mutation variants/genome edits (GE; indicated by orange and blue colours). **(B)** To establish stable genome-edited lines, mosaic F0 founders can either be crossed with wild-type (wt/wt) fish (upper panel) to obtain biallelic genome-edited offspring in the F2 generation (GE/GE), or crossed with other mosaic founders (lower panel) to generate biallelic genome-edited offspring already in the F1 generation. Biallelic genome-edited individuals may carry either identical edited alleles (homozygous) or different edited alleles (compound heterozygous).

To date, most genome editing approaches in aquaculture species have focused on generating gene knock-out models. A number of knock-out models have been reported in Atlantic salmon, targeting genes involved in reproduction, such as *dnd1* (Wargelius et al., 2016), *fshr* (Andersson et al., 2024), *bmp15* (Crespo et al., 2024), *piwil1* (Almeida et al., 2022), and *stra8* (Skaftnesmo et al., 2021); pigmentation (Edvardsen et al., 2014; Raudstein et al., 2023); fatty acid synthesis (Datsomor et al., 2019); or immunity (Raudstein et al., 2025), as well as in other economically important fish species (Blix et al., 2021). Such studies have provided insights into gene function, and in some cases, demonstrated outcomes with potential breeding applications, including the production of sterile fish (Fujihara et al., 2022; Wargelius et al., 2016; Xu et al., 2024), mono-sex populations (Jiang et al., 2016), or enhanced growth performance (Khalil et al., 2017; Ohama et al., 2020). For example, genetically sterile (germ cell-free) Atlantic salmon *dnd1* mutants followed through a full production cycle showed a long-term performance similar to their wild-type counterparts (Kleppe et al., 2022). Some future applications in aquaculture may require more complex genetic modifications such as multiplex gene knock-out, or even knock-in, approaches. Knock-in of genetic material remains challenging with the CRISPR/Cas-based approaches explored so far in aquaculture fish. This challenge is largely due to the low frequency of the homology-directed repair (HDR) pathway compared to the competing non-homologous end joining (NHEJ) pathway. Nevertheless, some studies have reported the successful insertion of whole transgenes. For example, in channel catfish, the insertion of transgenes by CRISPR/Cas9-mediated engineering led to improved disease resistance (Wang et al., 2024) and higher fatty acid content (Xing et al., 2022). While small insertions and single nucleotide changes have been reported in Atlantic salmon (Raudstein et al., 2024; 2023; Straume et al., 2021), reliable insertion of larger genetic elements and loci remains challenging in this species.

Exploring alternative strategies for delivering CRISPR/Cas systems is essential to overcome current challenges and translate genome editing from research stage into scalable tools for improving genetic gain in aquaculture. For example, in model fish species such as the Japanese rice fish (a.k.a. medaka), a GSC transplantation-based approach has been established to mass-produce genetically sterile (*fshr* gene-edited) fish (Nagasawa et al., 2019). Similar approaches have also been developed in other animal models. In avian species, researchers have combined primordial germ cell (PGC; precursors of GSCs) culture, genome editing *in vitro*, and transplantation protocols to generate both knock-out and knock-in models of interest (Hagihara et al., 2020; Ho Ching Chan et al., 2025; Taylor et al., 2017; Ballantyne et al., 2021). Transfer and adaptation of knowledge from other animal models may therefore provide a framework for developing similar working technologies in aquaculture fish species. The future possibilities and challenges of combining genome editing with GSC culture and subsequent transplantation will be discussed following a brief description of germline stem and progenitor cells and surrogate broodstock technology.

## 3 Brief description of germline stem and progenitor cells, *in vitro* culture, and transplantation in teleosts

### 3.1 Germline stem and progenitor cells and their *in vitro* culture

Germline development encompasses multiple cellular stages, from primordial germ cells (PGCs) specified during embryogenesis, to sexually differentiated germline stem cells (GSCs) and mature gametes. In this review, we primarily focus on the two germline cell populations that possess stem cell properties: PGCs and GSCs. PGCs are undifferentiated precursors of germline cells, specified during early embryogenesis by maternally deposited germplasm factors (Yamaha et al., 2010). Following specification, PGCs migrate via chemotactic signals to colonise the developing genital ridges, where they proliferate and associate with supporting somatic cells as part of a defined anatomical niche. After colonisation and subsequent sexual differentiation, a subset of PGCs become the adult GSCs (undifferentiated oogonia and spermatogonia in female and male, respectively). These cells can either self-renew or enter oogenesis and spermatogenesis to ultimately produce mature gametes. Detailed descriptions covering all aspects of germ cell development and differentiation in fish are provided elsewhere (Schulz et al., 2024; Zhao et al., 2024).

Culturing germ cells *in vitro* requires the isolation and enrichment of a homogeneous population of cells. Obtaining embryonic PGCs is technically challenging due to their low abundance and the small size of the developing genital ridges. As a result, fluorescence-based approaches are typically required to be able to identify and specifically recover this cell population. Early work by Yoshizaki and colleagues pioneered the use of a transgenic rainbow trout model expressing green fluorescent protein driven by germ cell-specific gene regulatory elements (i.e., *vasa* 3′UTR), enabling identification of PGCs (Yoshizaki et al., 2000). Transient labelling approaches based on microinjection of reporter mRNAs have also been used successfully (Nagasawa et al., 2013; Saito et al., 2006). Despite the advances in identifying and isolating PGCs from fish, maintaining and propagating them in culture conditions over an extended period has proven difficult. With the exception of a 4-month culture method reported in the model species zebrafish (Fan et al., 2008), long-term, stable PGC culture systems remain practically non-existent in fish. Adult GSCs, on the other hand, are generally easier to isolate due to their higher cell number and the larger size of gonadal tissue in sexually differentiated fish. Several studies have reported short- to medium-term culture of male spermatogonial cells (including GSCs) in different model and aquaculture fish species (Poursaeid et al., 2020; Shikina et al., 2008; Tan et al., 2023; Xie et al., 2022; Zhou et al., 2025). Although these systems are probably best described as primary cell cultures rather than continuously maintained cell lines, efforts to further improve GSC self-renewal, proliferation, and maintenance have included supplementation of fish-specific serum and/or embryonic extract, and recombinant human growth factors such as fibroblast growth factor (FGF), leukaemia inhibitory factor (LIF), or glial cell line-derived neurotrophic factor (GDNF) (Hong et al., 2004; Iwasaki-Takahashi et al., 2020; Zhou et al., 2025). Additionally, gonadal somatic cell feeder layers have been used to more closely resemble the *in vivo* gonadal niche that provides the microenvironment where GSCs interact with somatic supporting cells, and have shown a clear impact on GSC propagation (Iwasaki-Takahashi et al., 2020; Poursaeid et al., 2020; Xie et al., 2022). A recent review provides a broader overview of progress in fish GSC cell cultures, including the species and cell types investigated and applications such as germ cell transplantation (Ren et al., 2026).

### 3.2 Germ cell transplantation: surrogate broodstock technology

Surrogate broodstock technology (SBT) involves the production of donor-derived gametes and offspring through germ cell transplantation into sterile or germ cell-depleted recipient fish (the surrogates). In salmonids, the most commonly used SBT approach involves the preparation of gonadal cell suspensions (containing GSCs) extracted from gonadal tissue of immature donor individuals of interest, followed by transplantation into sterilised surrogates at an early life stage (around hatching) (Kleppe et al., 2025; Yoshizaki and Lee, 2018). Subsequently, the GSCs migrate to and colonise the forming genital ridges, where they are supported by the gonadal somatic cells of the surrogate. This somatic microenvironment supports donor GSC propagation, differentiation, and eventually maturation into functional donor-derived gametes carrying the genetics of the donor individual(s). Interestingly, in fish species such as Atlantic salmon, rainbow trout, masu salmon, and Japanese flounder, the fate of transplanted GSCs is determined by the somatic microenvironment of the surrogate; female and male surrogates produce eggs and sperm respectively, regardless of the sex of the donor fish (Okutsu et al., 2006; Yoshizaki et al., 2010; Lee et al., 2021; Ren et al., 2021; Kleppe et al., 2025). Thus, in these species, GSCs derived from either sex can differentiate into both male and female gametes, increasing the flexibility and efficiency of donor-derived fish production. Importantly, GSCs from either sex can be cryopreserved prior to transplantation and subsequently give rise to functional gametes (Yoshizaki and Lee, 2018). In contrast to conventional practices, where only male genetic material is routinely preserved via sperm cryopreservation due to the poor cryotolerance of eggs, SBT enables long-term storage of both female and male genetic resources, thereby increasing the range of genetic diversity that can be stored and later utilised.

SBT, including species-specific methodologies for germ cell preparation, transplantation and gamete production, surrogate preparation (sterilisation), factors influencing efficiency (such as donor/surrogate type, cell concentration, timing, and route of transplantation), as well as its many practical applications in aquaculture and conservation of endangered species, is extensively reviewed elsewhere (Goto and Saito, 2019; Jin et al., 2021; Kumar et al., 2026; Ryu et al., 2022; Yoshizaki and Yazawa, 2019). Briefly, among its applications within aquaculture is the potential for mass production of genetically sterile fish. Sterility is a highly desirable trait in farmed fish in order to prevent genetic introgression into wild populations following escape events, and it also mitigates the negative effects on health and growth associated with precocious sexual maturation (Taranger et al., 2010). However, sterility is inherently a challenging trait to pass on from a broodstock population, especially when it results from the absence of germ cells (such as in *dnd1* knock-out fish). An alternative strategy is to induce sterility while preserving the germline lineage, for example, by disrupting factors that are required for entry into puberty. In medaka, disruption of the *follicle-stimulating hormone receptor* (*fshr*) gene, expressed in the somatic gonadal supporting cells, results in sterile females with underdeveloped ovaries, while the males develop normal testes and produce functional sperm (Murozumi et al., 2014). Building on this phenotype, Nagasawa and colleagues transplanted GSCs from XX *fshr*-edited sex-reversed males into non-edited hybrid sterile female recipients that retained a functional somatic gonadal environment (Nagasawa et al., 2019). These surrogates proceeded with gametogenesis and produced functional donor-derived eggs carrying the *fshr* mutation. Subsequent crossing with XX *fshr*-edited sex-reversed males resulted in all-female sterile offspring. In salmonids, recent studies have shown that *fshr* knock-out Atlantic salmon males (females were not examined) (Andersson et al., 2024), and male and female rainbow trout–similar to female medaka–are sterile (as mentioned in Hayashi et al., 2026). Therefore, GSCs carrying a *fshr* knock-out mutation could potentially be transplanted into sterile male and female recipients with a functional somatic gonadal environment, enabling upon mating the mass-production of 100% sterile offspring without having to induce sex reversal or produce mono-sex populations.

## 4 Discussion

Genome editing combined with GSC transplantation technology has the potential to be a transformative platform for introducing precise genetic changes in aquaculture fish species. Realising this potential, however, requires efficient methods for generating genome-edited GSCs suitable for transplantation. Currently, such cells would most likely be obtained from established and validated genome-edited fish lines carrying defined genetic mutations. As mentioned, generating these mutant lines in Atlantic salmon requires substantial time and effort due to the long generation time. A more direct and refined approach would be to edit, propagate, and validate GSCs *in vitro* prior to transplantation (Figure 2). One strategy would involve isolating GSCs from wild-type fish, performing genome editing *in vitro*, selecting successfully edited cells, and subsequently transplanting them into sterile surrogate recipients. Alternatively, GSCs could be isolated from genome-edited F0 mosaic founders, thereby bypassing *in vitro* editing, although this approach would still require the initial production of F0 fish through conventional microinjection. In both scenarios, robust culture systems capable of supporting GSC expansion would be essential to generate sufficient cell numbers and enable screening and validation of the intended genetic edits. Transplantation of validated GSCs into multiple sterile (preferably germ cell-free) male and female surrogate fish could potentially enable the large-scale production of gametes carrying identical genetic mutations. Overall, the integration of genome editing, GSC culture, and transplantation technologies has the potential to greatly improve scalability and reduce the time required to establish genome-edited fish lines compared with conventional approaches based on embryo microinjection and multigenerational breeding. Such advances may be particularly valuable for non-model aquaculture species.

**FIGURE 2:**
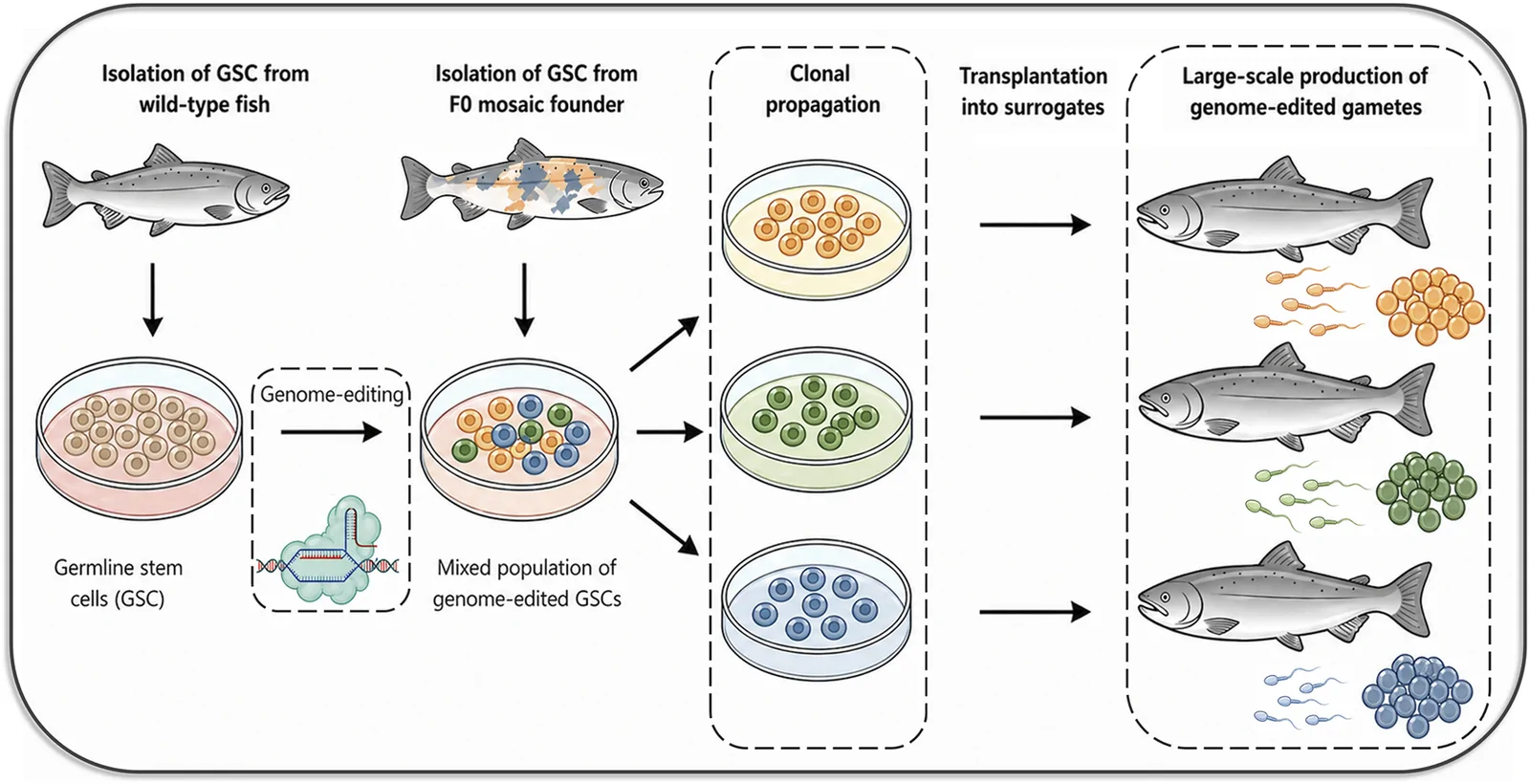
Proposed genome editing workflow using *in vitro* cultured germline stem cells (GSCs) from Atlantic salmon. GSCs could be isolated either from wild-type fish and genome-edited *in vitro*, or directly isolated from previously genome-edited F0 mosaic founders. Following genome editing, individual GSCs could potentially be clonally propagated, and resulting clones screened to identify those carrying the intended edit. Validated GSC clones could then be transplanted into sterile surrogate fish, which are germ cell-free and cannot produce their own offspring. Successful gonadal colonisation and donor-derived gamete production would be required for subsequent generation of gametes carrying the validated genetic mutation. While wild-type GSC isolation and transplantation have been demonstrated in wild-type Atlantic salmon, the dashed boxes indicate steps that remain to be established, including GSC culture systems, *in vitro* genome editing protocols, clonal expansion, and maintenance of germline competence of the edited GSCs following culture.

Despite the potential of these technologies, several challenges remain for the establishment of robust germ cell culture systems for aquaculture species. One key challenge is the isolation and enrichment of germ cells in sufficient numbers for the downstream applications. PGCs are present in low numbers during early development (Nagasawa et al., 2013; Saito et al., 2006) and their recovery typically requires dissection of small embryos. A possible strategy for increasing the number of PGCs available for isolation is the emerging technology of inducing the generation of PGC-like cells (iPGCs). Here, embryonic cells are directed towards a germline fate through microinjection of germline determinants *in vivo (*e.g., *dnd1* and *nanos3*). However, this approach has so far only been reported in fish model species (Nishimura and Fujimoto, 2025; Wang et al., 2023). On the other hand, GSCs from juvenile or adult fish are easier to obtain due to the larger size of the gonads and the higher germ cell abundance. Identification and isolation of transplantable germ cells can be achieved using methods such as fluorescence-activated cell sorting in combination with transgenic reporter systems for PGCs (Kobayashi et al., 2004) or magnetic-activated cell sorting using type A spermatogonia-specific antibodies (Ichida et al., 2019). However, such tools are currently only available for a limited number of aquaculture species. A further challenge is the development of defined culture conditions that support germ cell survival and proliferation *in vitro*. Although several studies have found improved culture conditions for fish GSCs, no universal protocol exists, and requirements for growth factors and hormones appear to be highly species-specific (Xie et al., 2020). Accordingly, optimal conditions, including medium composition, osmolality, temperature and supplement combinations, must therefore be determined for each species individually. A further limitation is the relatively restricted availability of fish-specific molecular tools and recombinant proteins compared with mammalian systems, which benefit from decades of biomedical research. Nevertheless, multiple studies have demonstrated that somatic cell feeder layers, which more closely mimic the gonadal niche, can significantly improve GSC survival and proliferation across different fish species (Iwasaki-Takahashi et al., 2020; Poursaeid et al., 2020; Xie et al., 2022). Looking forward, identifying and producing appropriate fish-specific recombinant growth factors and hormones is likely to be critical for establishing stable GSC cultures in aquaculture species. One promising strategy for identifying such factors is to apply emerging omics technologies such as single-cell RNA sequencing or spatial transcriptomics to elucidate specific cell-cell signalling pathways that mediate interactions between somatic supporting cells and GSCs within the gonads.

Once culture conditions are established, genome editing protocols specifically optimised for GSCs will need to be developed. CRISPR-based genome editing has been demonstrated in fish somatic cell lines using electroporation and lentiviral transduction approaches (Dehler et al., 2016; Gratacap et al., 2020a; 2020b; Wang et al., 2026). These studies may serve as a basis for the development of GSC-specific delivery protocols, although it remains unclear whether, and to what extent, cultured GSCs will respond to these methods. A recent study investigated the effect of various transfection and electroporation approaches on primary GSCs obtained from ricefield eel and reported successful *in vitro* genome editing of the cells (Zhan et al., 2026). Nonetheless, the study did not assess whether the edited cells could be clonally expanded and selected based on the desired edits, nor was subsequent transplantation performed and therefore germline competence and successful transmission of the edits have not yet been confirmed. Once successfully established for a given species, *in vitro* genome editing of GSCs may provide an opportunity to optimise precise knock-in strategies prior to transplantation. For relatively small genetic alterations, such as the introduction of nucleotide changes or small inserts, HDR may provide a means of generating precise edits. In contrast, insertion of larger genetic elements, such as reporter genes or transgenes, may represent additional challenges, including low HDR efficiency, delivery-associated toxicity, random donor integration, or unintended structural alterations at the target locus. Unlike embryo microinjection, *in vitro* culture allows repeated testing of editing parameters, including donor template design, delivery conditions, and potentially manipulation of DNA repair pathway activity to favour HDR. Approaches that have improved knock-in efficiency or reduced unwanted integration in somatic cells or other vertebrate systems may provide useful starting points, but their potential effectiveness would need to be validated in fish GSCs.

Any *in vitro* genome editing would initially generate a mixed population of edited and unedited cells (mosaicism), similar to that observed following conventional genome editing via embryo microinjection. The same limitation applies if using GSCs from F0 mosaic individuals. Consequently, transplantation of such heterogeneous cell populations into sterile surrogates could result in a mosaic population of gametes. One potential solution would be the isolation and expansion of single-cell clones from the mixed cell population. This would allow screening and validation of the target locus in monoclonal GSC populations, including confirmation of the intended edit, absence of unintended on-target alterations, and assessment of potential off-target changes and chromosomal stability before transplantation. Clonal expansion has been achieved in somatic cell lines from Chinook salmon (Collins et al., 2024) and rainbow trout (Zoppo et al., 2021). However, as fish GSCs are generally difficult to propagate, expanding them as single clones may be particularly challenging. It is also worth noting that using a limited number of clones for gamete production could reduce genetic diversity, and breeding programmes may therefore need to incorporate multiple independently edited clones and/or genetic backgrounds to maintain the diversity.

Ultimately, the potential scalability of this proposed approach will depend on several factors that are yet to be established in Atlantic salmon. As a starting point, a sufficient number of GSCs must be isolated and expanded while retaining their capacity to colonise the genital ridges of recipients following transplantation, as demonstrated in zebrafish (Wong et al., 2013), Nile tilapia (Lacerda et al., 2013) and rainbow trout (Shikina et al., 2008; Iwasaki-Takahashi et al., 2020). Whether the GSCs will be isolated from wild-type fish for subsequent *in vitro* genome editing or from genome-edited F0 mosaic fish, recovery and verification of correctly edited cells (including establishment of culture conditions and clonal expansion) will determine the number of validated cells available for transplantation. The effects of prolonged *in vitro* culture, genome editing, and clonal expansion, on GSCs and their germline competence remain unknown, including the ability of GSCs to colonise recipient gonads, and subsequent development into functional gametes. While successful transplantation of non-cultured wild-type GSCs has been demonstrated in Atlantic salmon (Kleppe et al., 2025), transplantation efficiency has so far remained low. Therefore, protocol optimisation is required to achieve higher transplantation success, and it remains to be established whether cultured and genome-edited GSCs can retain comparable or improved capacity for gonadal colonisation.

Insights into how some of these challenges may be addressed could be gained from studies in other animal groups. For example, porcine spermatogonial cells have been successfully genome-edited *in vitro*, although long-term culture systems remain limited (Goldsmith et al., 2022; Webster et al., 2020). In chickens, where one-cell embryo microinjection is not feasible, researchers have established long-term cultures of PGCs that can be edited *in vitro* and subsequently transplanted into recipient embryos (Whyte et al., 2015). As avian PGCs migrate through the bloodstream during embryonic development, they can be isolated directly from embryonic blood, edited, expanded in culture, and specifically selected before being injected into the blood circulation of sterile surrogate embryos, where they colonise the developing gonads. This strategy has enabled the generation of non-mosaic, genetically modified chickens through germline transmission (Ballantyne et al., 2021; Taylor et al., 2017). Although the reproductive biology of birds and mammals differ from fish, these studies provide a useful conceptual framework for developing similar strategies in aquaculture fish. One notable advantage of fish is their high fecundity, with many aquaculture fish species producing thousands of eggs in a single spawning event. If efficient GSC culture and transplantation systems can be established for fish, this could potentially represent an especially powerful and scalable approach for precision breeding and the dissemination of desirable genome-edited traits in finfish aquaculture.

## 5 Conclusion

Genome editing has the potential to complement current selective breeding programmes by accelerating genetic gain in aquaculture fish species, for example, through the precise introduction of disease resistance traits. While a small number of genome-edited fishes have been approved for human consumption, the broader implementation of genome editing in aquaculture remains limited. This is partly due to regulatory considerations, but also to several technical challenges associated with current genome editing approaches in fish.

The establishment of robust species-specific GSC culture and transplantation protocols may provide an alternative platform for implementing genome editing in finfish aquaculture. In particular, these methodologies offer the potential to improve scalability, reduce mosaicism, and shorten breeding timelines. Although technical challenges remain, advances in other animal groups demonstrate the feasibility of using GSC-based technologies to generate genome-edited individuals. Given the high fecundity of many fish species, similar approaches in aquaculture could potentially enable the efficient production of large numbers of genome-edited gametes. Continued development of GSC technologies, particularly culture systems that support the propagation and clonal expansion of genome-edited GSCs, may represent an important step towards the application of genome editing to accelerate genetic gain, and improve fish health and welfare in aquaculture.
